# The Effect of Self-Discrepancy on Online Behavior: A Literature Review

**DOI:** 10.3389/fpsyg.2022.883736

**Published:** 2022-04-26

**Authors:** Chuan Hu, Rui Cao, Jiao Huang, Ying Wei

**Affiliations:** ^1^School of Public Policy and Administration, Nanchang University, Nanchang, China; ^2^School of Information Management, Jiangxi University of Finance and Economics, Nanchang, China

**Keywords:** self-discrepancy, self-concept, user behavior, literature review, Internet

## Abstract

People will experience self-discrepancy when they perceive their actual self is distanced from their ideal self and ought self. The perceived self-discrepancy has a significant impact on people’s online behavior. The goal of this paper is to provide an overview of the research on self-discrepancy in the Internet era through a literature review. A total of 37 papers were included in this review. First, this study presents the basic characteristic of the reviewed articles (such as research contexts, samples, and data collection methods). Second, it summarized how the Internet affected people’s self-discrepancy. Third, this review identified how self-discrepancy was associated with different kinds of online behavior (such as impression management, knowledge contribution, and gaming addiction). Finally, this study outlined several possible directions for future research.

## Introduction

The self-discrepancy theory proposes that people’s behavior is motivated to reduce the self-discrepancy between the self that they actually present and the self they ought or wish to be (Higgins, 1987). Existing studies have explored the relationships between different types of self-discrepancies and people’s behavior from different perspectives in various contexts, such as photo editing behavior on social network sites (Mankotia and Wesley, 2020; Beos et al., 2021; Stewart and Clayton, 2021; Yang et al., 2021), knowledge contribution behavior in online communities (Suh, 2013; Lee and Suh, 2015), and gaming behavior in online games (Kim M.-K. et al., 2018a; Mancini et al., 2019; Yang et al., 2021).

However, prior studies are quite fragmented and diverse. Although some researchers have reviewed a few articles related to self-discrepancy, they focused on some specific perspectives, including consumer behavior (Mandel et al., 2017), wellbeing (Kelly et al., 2015), and body dissatisfaction among individuals with eating disorders (Lantz et al., 2018). These studies paid more attention to human behavior in the offline world. The emergence of the Internet provides people with a free environment for strategical self-presentation, which in turn may have significant impacts on their self-discrepancy and behavior (Bizman et al., 2001; Hu et al., 2015; Ahadzadeh et al., 2017; Huang et al., 2019). Therefore, a comprehensive and rigorous review of the existing literature on the effect of an individual’s self-discrepancy on online behavior is still called for.

The current study aims to fulfill the above-mentioned gap by reviewing relevant articles and answering the following research questions: (1) how does the Internet affect people’s self-discrepancy? (2) What kinds of online behavior are associated with self-discrepancy?

## The Concept of Self-Discrepancy

### Self-State Representations, Self-Discrepancy, and Behavior

In the last century, many psychologists tried to identify different facets of an individual’s self-concept, such as two actual selves from the perspective of individuals themselves and others (Erikson, 1959; Wylie, 1979), spiritual self and social self (Pedersen, 2000), possible selves and now selves (Markus and Nurius, 1986; Leung, 2011), and so on. It is a long-accepted notion that an individual’s self-concept consists of different self-state representations. Higgins (1987) integrated different facets of self-concept proposed by previous theorists and proposed self-discrepancy theory, which provides a systematic and comprehensive framework to reveal the interrelations among different self-states.

Self-discrepancy theory (Higgins, 1987, 1989) postulates three basic domains of the self (i.e., actual self, ought self, and ideal self) and two basic standpoints on the self (people’s own personal standpoint and the standpoint of some significant others). More specifically, the actual self represents the attributes that individuals or their significant others believe they actually are. The ideal self and the ought self represent the attributes that individuals or their significant others think they can ideally possess and ought to possess, respectively. In addition, the combination of the ideal self and the ought self was defined as an individual’s self-guide, which is the standard for self-evaluation and self-enhancement in daily life (Higgins, 1987). Self-discrepancy refers to the gap between the actual self and the self-guide. According to self-discrepancy theory, the greater an individual’s self-discrepancy is, the more discomforts he or she will suffer (Higgins, 1987, 1989). It is suggested that people’s behavior is oriented to reduce self-discrepancy (Hu et al., 2015), thereby relieving discomfort and negative emotions.

Individuals may present themselves differently in different contexts (Gee, 2000; Marwick, 2013). The phenomenon that people selectively present themselves in different situations was defined as “strategical self-presentation” (Ranzini and Lutz, 2017; Jang et al., 2018). With the behavior of strategical self-presentation, people can reduce their self-discrepancy to some extent. For instance, an unattractive person can present a better self-image and reduce actual-ideal self-discrepancy by wearing fashionable clothes.

### Virtual Self-Discrepancy on the Internet

The development of various online platforms (such as social network sites and online games) allows people to present themselves strategically to a greater extent. Individuals can construct their identity in virtual spaces and create a virtual self-online freely (Suh, 2013). An individual’s virtual self is likely to be different from the actual self. For example, on social network sites, people may manipulate their photos to build a more desirable image. In online games, players can pick or construct different game avatars (Loewen et al., 2021). It is found that players perceive their game avatars as more extroverted and less neurotic (Bessière et al., 2007). People also rate the virtual avatar in Second Life as more outgoing and attractive than themselves (Messinger, 2007). Scholars proposed the concept of virtual self-discrepancy to describe the degree to which an individual’s virtual identity deviates from the individual’s actual identity in the real world (Jin, 2012). Individuals may experience virtual self-discrepancy when they engage in online activities. Previous studies suggested that virtual self-presentation has a significant influence on people’s emotions and behavior. Thus, the current study will review the effect of both traditional self-discrepancy and virtual self-discrepancy on online behavior.

## Methodology

### Search Strategy

To identify studies relevant to the effect of self-discrepancy on online behavior, five major databases were used in the literature search phase, including Scopus, ScienceDirect, Web of Science, Academic Search Complete, and Wiley Online Library. These databases cover a wide range of disciplines and a large number of peer-reviewed journals. In the search process, the terms related to self-discrepancy (such as “self-discrepanc*,” “actual-ideal,” and “actual-ought”) were combined with terms related to the online context (such as “online,” “cyber,” “Internet,” and “virtual”) as keywords. Full-text searches were conducted to cover as many studies as possible. The search was conducted in December 2021. There was no limitation on the time interval of the publications. We searched for possibly relevant articles published by the date of the literature search, yielding a total of 2,570 papers.

### Selection Process

To select relevant articles from the search result, a set of eligibility criteria were established. Studies were included if they meet all the following inclusion criteria: (a) investigate the effect of self-discrepancy; (b) involve an online context; (c) published in peer-reviewed journals; and (d) written in English. The exclusion criteria ensure that studies were not (a) conference papers, book chapters, and review papers; (b) only focusing on the offline context. Both qualitative and quantitative studies were considered in this review as long as they examined the effect of self-discrepancy.

**Figure 1:**
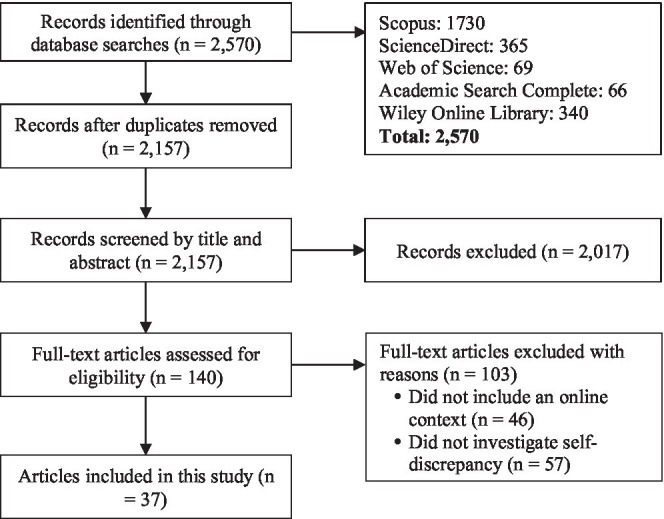
Literature selection flowchart.

After removing duplicates across databases, 2,157 articles were left. Next, the titles and abstracts of the studies were checked. Most studies (*n* = 2,017) were removed based on the inclusion and exclusion criteria. Then, the authors went through the full text of the remaining 140 articles to access their relevance based on the eligibility criteria and research questions. One hundred three studies were further excluded. Finally, 37 articles were included for this review. Figure 1 presents the flowchart of the selection process.

## Results

### Basic Characteristics of the Studies

Table 1 presents a summary of the basic characteristic of the reviewed articles. As shown in Table 1, a total of 13 studies (35.1%) used a student sample. Three studies (8.1%) used a mixed sample of students and non-students. The remaining 21 studies (56.8%) used a non-student sample or did not illustrate the characteristics of their participants clearly. The sample size ranged from 33 to 1,063.

**Table 1:** Basic characteristic of reviewed articles.

Authors	Data collection method	Sample	Research context	Theory	Category
Ahadzadeh et al. (2017)	Survey	273 university students	SNS (Instagram)	Self-discrepancy theory, social comparison theory, self-schema theory	Impression management behavior
Beos et al. (2021)	Survey	227 Australian women	General SNS	N/A	Impression management behavior
Bessière et al. (2007)	Survey	51 WoW players (university students and members from a gaming group)	Online games	Self-discrepancy theory	Avatar creation behavior
Dengah and Snodgrass (2020)	Experiment and interview	78 participants	Videogame	Self-discrepancy theory	Avatar creation behavior
Dunn and Guadagno (2012)	Experiment and survey	174 university students	Online games	Self-discrepancy theory, Big Five Personality, Self-presentation theory	Avatar creation behavior
Flynn et al. (2020a)	Survey	277 adult men	General SNS	Self-discrepancy theory	Impression management behavior
Flynn et al. (2020b)	Survey	253 adult women	General SNS	Self-discrepancy theory	Impression management behavior
Hu et al. (2015)	Interview and survey	47 community members	Social network communities	Self-discrepancy theory, regulatory focus theory	Impression management behavior
Hu et al. (2017)	Interview and survey	57 participants	SNS (QQ)	Self-discrepancy theory, self-determination theory	Impression management behavior
Hu et al. (2020)	Survey	837 QQ community members	Social network communities	Self-discrepancy theory	Impression management behavior
Huang et al. (2019)	Survey	1,063 respondents	Social network communities	Self-discrepancy theory, regulatory focus theory	Impression management behavior
Jin (2012)	Experiment and survey	Study 1: 117 undergraduates Study 2: 148 undergraduates	Second life app	Expectancy violation theory, uncertainty reduction theory, and social penetration theory	Avatar creation behavior
Johnson and Ranzini (2018)	Experiment	168 adults	SNS (Facebook)	Self-discrepancy theory	Impression management behavior
Kim and Damhorst (2010)	Survey	348 college female students	Online shopping	Self-discrepancy theory	Commercial behavior
Kim and Kim (2016)	Survey	223 undergraduate students	SNS (Facebook)	N/A	Commercial behavior
Kim M.-K. et al. (2018a)	Experiment	39 participants	Online games	Self-discrepancy theory	Addiction behavior
Kim T.-H. et al. (2018b)	Survey	33 middle school students	Internet and smartphone	N/A	Addiction behavior
Kirkpatrick and Lee (2021)	Experiment	309 participants	SNS (Instagram)	Self-discrepancy theory, social comparison theory	Impression management behavior
Lee and Suh (2015)	Survey	300 virtual community members	Online communities	Theory of psychological ownership	Knowledge contribution behavior
Lee et al. (2014)	Survey	238 respondents from an online research company	Online communities	Self-discrepancy theory, the stimulus-organism-response (S-O-R) framework, self-presentation theory	Knowledge contribution behavior
Li et al. (2011)	Survey	161 students (13–15 years old)	Online games	Self-discrepancy theory	Addiction behavior
Li et al. (2019)	Survey	310 members of Qzone	SNS (Qzone)	Self-discrepancy theory, symbolic self-completion theory	Commercial behavior
Liu et al. (2020)	Survey	641 WeChat users	SNS (WeChat)	Self-discrepancy theory	Impression management behavior
Loewen et al. (2021)	Survey	125 undergraduate gamers	Online games	Self-discrepancy theory	Avatar creation behavior
Mancini et al. (2019)	Survey and experiment	Study 1: 770 players Study 2: 100 participants	Online games	Self-discrepancy theory	Addiction behavior
Mankotia and Wesley (2020)	Survey	106 females (students and employees)	Social media	Self-discrepancy theory	Impression management behavior
Meng et al. (2015)	Experiment	247 undergraduate female students	YouTube	Self-discrepancy theory	Impression management behavior
Pimentel and Kalyanaraman (2020)	Experiments	Experiment 1: 90 participants Experiment 2: 122 participants	Digital games	Self-discrepancy theory	Avatar creation behavior
Rabbanee et al. (2020)	Survey	Study 1: 282 students Study 2: 343 respondents	SNS (Facebook)	Self-discrepancy theory, attachment theory	Commercial behavior
Shin (2021)	Survey	515 respondents	Online shopping	Self-discrepancy theory, the theory of planned behavior	Commercial behavior
Siibak (2009)	Survey	713 students (11–18 years old)	SNS (Rate)	Self-discrepancy theory	Impression management behavior
Stewart and Clayton (2021)	Survey	264 Instagram users	SNS (Instagram)	Self-discrepancy theory	Impression management behavior
Suh (2013)	Survey	299 respondents from an Internet survey company	Online communities	Self-discrepancy theory, social cognitive theory	Knowledge contribution behavior
Tzeng (2010)	Survey	311 college students	Online world	Self-discrepancy theory	Addiction behavior
Li et al. (2021)	Survey	508 university student gamers	Online games	Self-discrepancy theory	Addiction behavior
Yang et al. (2021)	Survey	443 (196 United States college females and 247 Chinese college females)	Social media	Self-discrepancy theory, dramaturgical theory	Impression management behavior
Yu and Kim (2020)	Survey	299 female people	SNS (Facebook)	N/A	Impression management behavior

*SNS, Social network sites; N/A, Not applicable*.

Regarding the research context, 14 articles were conducted on social network sites. Most of them investigated a specific site, such as Facebook, Instagram, and QQ. Nine studies were carried out in the context of online games. Six articles focused on self-discrepancy in online communities. Other research contexts include online shopping, social media (such as YouTube), the general online world, and so on.

Most reviewed articles used quantitative approaches. Only two studies adopted a qualitative research design. The most frequently used research methods are surveys and experiments. Experiments are often used in research conducted in the context of social network sites and online games. Scholars designed different experiments to investigate how self-discrepancy is associated with social network use and gaming behavior.

When it comes to the measurement of self-discrepancy, there are no fixed measurements. The instruments used in the reviewed articles can be classified into two categories: direct measurement and indirect measurement. Some studies assessed participants’ self-discrepancy directly. They used general items to evaluate participants’ overall perception of the discrepancy between different self-concepts (e.g., “In this online community, I am smarter and more intelligent than I am in the real world”). Some studies assessed participants’ self-discrepancy indirectly. Given that different people have different actual self and self-guide, some studies asked the participants to report different aspects of their self-concepts. Then, self-discrepancy was measured with the differences in scores. When using the indirect measurement, some research asked the participants to think about adjectives to describe their self-concept by themselves, while some other research provided a list of personality traits or description sentences based on the research themes and asked the participants to rate the items in the list from different perspectives (e.g., the extent to which the item reflects their ideal self, virtual self, or actual self).

### The Effect of the Internet on Self-Discrepancy

The emergence of the Internet has changed the way people express themselves and interact with others (Huang et al., 2019), which may affect people’s self-discrepancy on the Internet. Individuals may take advantage of the characteristics of the Internet and present themselves selectively online. Additionally, individuals can access various information and communicate with different people easily in the online world. The new information and new social connections online may influence their self-guide. In this case, the Internet may affect people’s self-discrepancy in the online world by changing the way people present their self-concept and the formations of their self-guide.

#### The Expression of Self-Concept

On the Internet, individuals can create a virtual self (Suh, 2013). When compared with face-to-face communications, people can present themselves more freely online after hiding physical characteristics behind the screen. Due to the anonymity of the Internet, people can hide or even fake personal information when presenting themselves in the online world (Hu et al., 2015). It is difficult for service providers and other users to validate the information. Lee et al. (2014) found that anonymity is positively associated with virtual self-discrepancy. When individuals feel that their behavior in online communities cannot be linked to their real identity, they are more likely to present an online identity that is different from their real identity (such as being friendlier). Therefore, individuals can construct their virtual self strategically online. It is found that people may present themselves based on the ideal self as much as they can in cyberspace, especially on some highly anonymous online platforms (Hu et al., 2015). Bessière et al. (2007) indicated that players tend to create favorable game characters that are similar to their ideal selves. As a consequence, the virtual self an individual present online may be better than the actual self in the real world. Thus, the distance between the virtual self and the self-guide is smaller. People will experience a decrease in self-discrepancy when interacting with others using the virtual identity online.

#### The Formation of Self-Guide

In addition to the free expression of self-concept, the Internet also affects the formation of one’s self-guide (i.e., ideal self and ought self). On the one hand, the Internet can reduce ought self in one’s self-guide. The anonymity of cyberspace frees people from the restraints of the corporal body and physical environment (Donath, 2002; Kim et al., 2011). Individuals perceive a lower level of surveillance and feel less pressured by social norms online (Selwyn, 2008; Hu et al., 2017). For example, Suh (2013) suggested that individuals who experience a higher level of autonomy in online communities tend to break social norms and shed inhibitions to a greater extent. The anonymous online environment allows individuals to behave negatively with fewer concerns (Hu et al., 2017). Therefore, people may experience a decrease of actual-ought discrepancy due to perceived fewer duties and responsibilities on the Internet.

On the other hand, the connectivity of the Internet enables people to access different information and different social groups more conveniently than in the physical world. The various information and values people receive online may affect their self-guide, which may, in turn, affect their self-discrepancy. For example, it is found that exposure to ideal photos on social network sites and exposure to ideal, thin models in online advertisements make people more concerned about their body type (Kim and Damhorst, 2010; Ahadzadeh et al., 2017). People tend to compare themselves with the ideal images online and perceive a greater self-discrepancy. In addition, it is suggested that the ideal body shape preferred by social network site users has a significant impact on individuals’ assessment of their body parts (Flynn et al., 2020a,b). Young people consider the expectations of virtual peer groups very important. The youth endeavor to meet these expectations in order to become popular (Siibak, 2009). Therefore, when individuals consider the online ideal images as the ideal self and integrate the values of online groups into self-guide, their self-guide becomes more complex and more difficult to fulfill. In this case, individuals may experience greater self-discrepancy.

#### Factors Affecting an Individual’s Self-Discrepancy in General

Existing studies also looked into some personal factors that can influence individuals’ self-discrepancy on the Internet. Some researchers focused on the effect of personalities. For example, Dunn and Guadagno (2012) investigated the association between personality and avatar creation in online games. They found that people with a higher level of extraversion and openness personality tend to pick avatars with fewer discrepancies from themselves in video games (Dunn and Guadagno, 2012). Dengah and Snodgrass (2020) suggested that self-esteem is another trait that predicts avatar actualization in games. Players with high self-esteem have lower actual-avatar self-discrepancy. In the context of social media, neuroticism personality is positively associated with body image self-discrepancy (Yang et al., 2021). Neurotic individuals are more likely to perceive a greater actual-ideal discrepancy in body image. Apart from personalities, gender and culture can also affect individuals’ perception of self-discrepancy. For example, Kirkpatrick and Lee (2021) found that female users are more likely to experience greater self-discrepancy and feel dissatisfied with their body type when viewing thin photos on Instagram. Yang et al. (2021) suggested that female college students in China exhibit higher body image self-discrepancy than their American contemporaries.

### The Effect of Self-Discrepancy on an Individual’s Behavior

The self-discrepancy theory proposes that different types of self-discrepancies will result in different kinds of psychological discomforts, including dejection-related emotions (such as disappointment, dissatisfaction, and shame) and agitation-related emotions (such as fear, guilt, and self-contempt; Higgins, 1987). An individual’s behavior is oriented to align their self-concept with self-guide to avoid those negative emotions (Higgins, 1987). When experiencing negative emotions induced by self-discrepancies, some people may engage in non-deviant behavior and try to overcome these psychological discomforts through reducing self-discrepancy. By contrast, some may engage in deviant behavior and try to escape from these discomforts temporarily by ignoring self-discrepancy.

#### Non-deviant Behavior

##### Impression Management Behavior

Existing research suggested that self-discrepancy can lead to impression management behavior. Given that the comparison between actual body type and ideal images online induces self-discrepancy (Kirkpatrick and Lee, 2021), people tend to feel dissatisfied with their actual body type (Flynn et al., 2020b). For example, it is found that body-related self-discrepancy caused by online models is positively related to consumers’ body dissatisfaction (Kim and Damhorst, 2010). Ahadzadeh et al. (2017) also suggested that self-discrepancy mediated the relationship between Instagram use and body dissatisfaction.

When people perceive self-discrepancy and experience negative emotions, some people tend to edit their photos to reduce actual-ideal self-discrepancy and manage their self-image. For example, Yang et al. (2021) suggested that a higher level of body image self-discrepancy will lead to a stronger intention for editing portrait photos. Beos et al. (2021) also found that appearance-based actual-ideal self-discrepancy is positively associated with facial dissatisfaction and photo manipulation. Similarly, Stewart and Clayton (2021) indicated that Instagram users with greater actual-ideal self-discrepancy are more likely to manipulate their photos. Self-discrepancy is also positively associated with narcissistic self-presentation on Facebook (Yu and Kim, 2020).

However, there are some inconsistent results. Mankotia and Wesley (2020) suggested that the discrepancy between actual-ideal self and actual-ought self is not significantly associated with photo editing behavior. A possible explanation may be that photo editing can result in some negative consequences, such as judgment from peers and romantic relationship conflict (e.g., jealousy and argument; Johnson and Ranzini, 2018; Stewart and Clayton, 2021). In addition, the exaggeration of online self-image increases the incongruence between an individual’s actual self and the self he or she presented online. This self-discrepancy tends to have a negative impact on one’s subjective wellbeing. People may experience anxiety in online communications (Yu and Kim, 2020).

The expectations of others have a significant impact on individuals’ impression management behavior. Liu et al. (2020) found that the negative emotions (such as social interaction anxiety and disappointment) caused by actual-ideal and actual-ought self-discrepancy will lead to lurking intention in interactions on social network sites. People who failed to live up to different expectations tend to behave passively on social network sites. In addition, it is found that perceived self-discrepancy between actual and ideal-other self drives individuals to share less unique content on Facebook (Johnson and Ranzini, 2018). People share media content (e.g., songs and films) that is widely known by others to leave a good impression on others.

##### Knowledge Contribution Behavior

Self-discrepancy also has some influences on knowledge contribution behavior. It is found that virtual self-discrepancy was positively associated with the quality and quantity of information sharing (Lee et al., 2014). An individual with a greater discrepancy between the actual self and virtual self experiences a higher level of autonomy and recovery in online communities, which in turn predicts this individual’s quality and quantity of contribution in online communities (Suh, 2013). Self-discrepancy can also improve contribution quality through psychological ownership (Lee and Suh, 2015). The time and effort people spend to create an ideal self-image enhances the possessive feeling of their virtual identity in the community. Thus, the actual-virtual self-discrepancy is positively associated with psychological ownership of a virtual community, which in turn increases individuals’ satisfaction, self-esteem, and contribution quality (Lee and Suh, 2015).

##### Commercial Behavior

Some researchers have investigated the link between self-discrepancy and commercial behavior. It is suggested that self-discrepancy predicts individuals’ interaction with a brand on Facebook (Kim and Kim, 2016). Rabbanee et al. (2020) also found that self-discrepancy has a significant impact on people’s attachment with a brand, which in turn affects their liking, sharing, and commenting behavior about the brand. Meng et al. (2015) examined the role of self-discrepancy in the effectiveness of media campaigns. They found that actual-ideal body self-discrepancy has no significant impact on the perceived effectiveness of media campaigns in changing females’ attitudes about disordered eating behavior. In addition to customer attitude, self-discrepancy is also associated with online shopping. Individuals buy digital items online to reduce self-discrepancy, thereby enhancing their self-esteem (Li et al., 2019). It is found that self-discrepancy moderates the relationship between pandemic fear and online shopping intention (Shin, 2021). People’s concerns about the COVID-19 pandemic have a positive effect on their online shopping intention, while this positive relationship is stronger among those with greater self-discrepancy.

##### Avatar Creation Behavior

Previous studies suggested that self-discrepancy is closely related to avatar creation behavior. Loewen et al. (2021) found that self-discrepancy has a significant impact on avatar creation style. Individuals with greater actual-ideal self-discrepancy prefer idealized avatars, while those with greater actual-ought self-discrepancy prefer fantasy avatars (i.e., completely different from themselves). Moreover, virtual self-discrepancy (i.e., the discrepancy between actual self and avatar) is associated with gaming experience and psychological wellbeing. Bessière et al. (2007) found that players with lower psychological wellbeing are more likely to experience greater virtual self-discrepancy. They tend to create favorable game characters that are similar to their ideal self. However, virtual self-discrepancy can induce negative gaming experiences, such as negative experiences in self-presence. Jin (2012) found that the discrepancy between the actual self and the avatar in Second Life is negatively associated with self-presence and flow. Individuals with greater virtual self-discrepancy are less likely to experience the avatar as if it were their actual self and they are less likely to obtain optimal and enjoyable experience in Second Life. Pimentel and Kalyanaraman (2020) found that the construction of an avatar with undesirable aspects of the actual self (e.g., physical disabilities) significantly reduces anxiety, which means that players with lower virtual self-discrepancy will experience less anxiety.

#### Deviant Behavior

For the impact of self-discrepancy on deviant behavior, most studies have focused on the Internet and gaming addiction. For example, Tzeng (2010) suggested that college students’ self-discrepancy in Internet use symbolizes some psychological or behavioral dissonances. The enhancement of peer relationship skills can help to reduce the actual-ideal discrepancy in adolescents, which may, in turn, reduce their risk for Internet and smartphone addiction (Kim T.-H. et al., 2018b). In addition, Li et al. (2011) found that actual-ideal self-discrepancy has a direct impact on pathological gaming. Individuals may use games as a tool to escape from negative emotions caused by self-discrepancy (Kim M.-K. et al., 2018a). Mancini et al. (2019) found that the idealization of avatars directly increases players’ gaming addiction. The greater the virtual discrepancy the more likely an individual will be addicted to online games. Li et al. (2021) also indicated that actual-ideal self-discrepancy is a predictor of Internet gaming addiction. Individuals may engage in Internet games to escape from reality. Although some researchers failed to identify a direct connection between actual-ideal self-discrepancy and Internet gaming disorder, it is found that the individuals with gaming disorder evaluate both actual self and ideal self more negatively than their peers (Kim M.-K. et al., 2018a).

## Discussion

The current study reviewed articles related to the effect of individuals’ self-discrepancy on online behavior. We found that the Internet can affect individuals’ self-discrepancy in the online world by allowing individuals to present their self-concept freely and influencing the formation of their self-guide. Existing studies have mainly examined the effect of self-discrepancy on impression management behavior, knowledge contribution behavior, commercial behavior, avatar creation behavior, and gaming addiction behavior. Most studies have identified positive associations between self-discrepancy and the above-mentioned online behavior. Although existing research has investigated the effect of self-discrepancy on online behavior from different perspectives, there are some gaps in previous studies.

First, more efforts should be made to explore the effect of actual-ought discrepancy. Most of the reviewed studies have focused on actual-ideal self-discrepancy and the discrepancy between the actual self and virtual self (i.e., virtual self-discrepancy; e.g., Jin, 2012; Suh, 2013; Beos et al., 2021; Stewart and Clayton, 2021). The research on actual-ought self-discrepancy is rare. As suggested in self-discrepancy theory, the ought self is an important part of people’s self-guide (Higgins, 1987). The discrepancy between the actual self and ought self can lead to agitation-related emotions, such as anxiety and self-contempt (Higgins, 1987). Thus, it would be an interesting direction for future studies to investigate how people cope with the negative experience induced by actual-ought self-discrepancy.

Second, there is a lack of research on self-discrepancy from the standpoint of others. Existing studies mainly looked into individuals’ self-discrepancy from their personal standpoint, evaluating the discrepancy between different self-concepts from their own perspective. However, the expectations and beliefs of significant others also have important impacts on individuals’ behavior. The self-discrepancy theory suggested that ideal-other self and ought-other self are essential components of one’s self-guide (Higgins, 1987). It is found that young people value the expectations of their online friends and manage their online images accordingly (Siibak, 2009). More empirical studies are needed to provide a comprehensive understanding of how people’s behavior is affected by actual-ideal other self-discrepancy and actual-ought other self-discrepancy.

Third, opportunities exist to investigate the effect of self-discrepancy on deviant behavior. Existing studies suggested that self-discrepancy can lead to addictive behavior, such as gaming addiction and Internet addiction (Li et al., 2011, 2021; Mancini et al., 2019). People create ideal avatars in games and escape from reality (Li et al., 2021). Apart from gaming addiction, self-discrepancy is also likely to be associated with other kinds of problematic online behavior. For example, people may vent their negative emotions in an anonymous online environment when they experience actual-ideal self-discrepancy. But, it is not clear whether these negative emotions will lead to aggressive online behavior, such as cyberbullying. Thus, future research is suggested to pay more attention to the connection between self-discrepancy and other kinds of problematic online behavior.

Fourth, more efforts should be made to explore how self-discrepancy affects people’s behavior over time. Most of the reviewed articles used cross-sectional data. Only one study collected data with experiments that lasted for 8 weeks (Kim T.-H. et al., 2018b). There is a lack of understanding of the long-term effect of self-discrepancy. For example, longitudinal evidence is needed to examine whether sustaining self-discrepancy has negative impacts on people’s behavior and attitudes, such as work performance, psychological wellbeing, and life satisfaction.

Fifth, future studies are suggested to improve the diversity of samples. Over one-third of the studies in this review used a student sample. However, the self-discrepancy and online behavior of students may be different from other populations (such as employed workers and elderly people). The generalizability of the findings may be limited by the student sample. Therefore, it would be better for future studies to consider recruiting more variable participants, such as participants of different ages, with different occupations, and from different cultures. The diversity of samples across studies may generate more variable and comprehensive conclusions regarding the effects of self-discrepancy.

### Contributions and Limitations

The findings of this review should be evaluated in the context of limitations. This study only considered peer-reviewed articles written in English in the review process. Therefore, the findings of this study might not be comprehensive enough. Future reviews are suggested to include studies published in other venues (such as book chapters and conference proceedings) and articles written in other languages.

Although this review has some limitations, it contributes to the research on self-discrepancy in several ways. This study presents the basic characteristics of the included studies, such as sample characteristics, research context, and data collection method. It elaborates on the impact of the Internet on people’s self-discrepancy. It is found that people may experience a decrease in self-discrepancy when they present themselves in the way they want online. Individuals may perceive an increase in self-discrepancy when they integrate online information and values into their self-guide. In addition, this study summarizes the effects of self-discrepancy on different kinds of online behavior. Results of this review provide an empirical landscape of the research on self-discrepancy in the Internet era. Also, this review identifies some gaps in existing studies and provides several recommendations for future research.

## Author Contributions

CH contributed to the design of the study and wrote most part of the manuscript. RC contributed to the process of literature search, literature selection, and data analysis. JH contributed to the design of the study, data analysis, and revision of the manuscript. YW helped in revising the manuscript. All authors contributed to the article and approved the submitted version.

## Funding

This study is supported by the Education Department of Jiangxi Province (project number: GL20207 and JC20216), National Natural Science Foundation of China (project number: 72001097), and Nanchang University (project number: QN202022).

## Conflict of Interest

The authors declare that the research was conducted in the absence of any commercial or financial relationships that could be construed as a potential conflict of interest.

## Publisher’s Note

All claims expressed in this article are solely those of the authors and do not necessarily represent those of their affiliated organizations, or those of the publisher, the editors and the reviewers. Any product that may be evaluated in this article, or claim that may be made by its manufacturer, is not guaranteed or endorsed by the publisher.
